# Quantitative imaging–pathology correlation in nodular fasciitis and diagnostic discrimination from sarcoma

**DOI:** 10.1186/s13244-026-02424-z

**Published:** 2026-11-02

**Authors:** Junhui Yuan, Shaobo Fang, Dongqiu Shan, Guangguang An, Renzhi Zhang, Xuejun Chen

**Affiliations:** 1 Department of Radiology, The Affiliated Cancer Hospital of Zhengzhou University & Henan Cancer Hospital, Zhengzhou, China; 2 Department of Radiology, The First Affiliated Hospital of Fujian Medical University, Fuzhou, China; 3 Department of Radiology, Zhengzhou University People’s Hospital & Henan Provincial People’s Hospital, Academy of Medical Sciences, Zhengzhou University, Zhengzhou, China; 4 National Cancer Center/National Clinical Research Center for Cancer/Cancer Hospital, Chinese Academy of Medical Sciences and Peking Union Medical College, Beijing, China

**Keywords:** Fasciitis, Magnetic resonance imaging, Sarcoma, Soft tissue neoplasms, Diffusion magnetic resonance imaging

## Abstract

**Objectives:**

To investigate associations between key magnetic resonance imaging features of nodular fasciitis (NF) and quantitative histopathologic biomarkers, and to evaluate whether their integration provides complementary information for differentiating NF from soft tissue sarcoma (STS) in diagnostically equivocal settings.

**Materials and methods:**

This retrospective study included 128 patients with NF and 133 with STS who underwent preoperative 3-T MRI between May 2023 and June 2025. Two blinded musculoskeletal radiologists independently evaluated predefined imaging features, including fascial tail sign, T2 heterogeneity score, enhancement pattern, perilesional edema, and mean/minimum apparent diffusion coefficient (ADC). Histopathology quantified cellular density, collagen content, and myxoid degeneration. Imaging–pathology correlations were assessed using Spearman analysis and multivariable regression. Diagnostic performance of MRI-only, pathology-only, and combined models was evaluated using logistic regression, receiver operating characteristic (ROC), area under the curve (AUC) analysis, calibration, decision curve analysis, and bootstrap validation.

**Results:**

T2 heterogeneity correlated positively with myxoid degeneration (ρ = 0.52) and negatively with collagen content (ρ = −0.28) (*p* < 0.001). Mean ADC correlated inversely with cellular density (ρ = −0.63, *p* < 0.001). Mean ADC was significantly higher in NF than STS (*p* < 0.001). The combined model outperformed MRI alone (AUC, 0.96 vs 0.89) and demonstrated greater net clinical benefit. Bootstrap validation confirmed stable performance (mean AUC, 0.960; 95% confidence interval, 0.938–0.978).

**Conclusion:**

Imaging features of NF were associated with underlying stromal and cellular substrates. Integrating imaging features with quantitative pathology biomarkers may support radiology–pathology concordance assessment in selected diagnostically equivocal cases.

**Key Points:**

***Question*** How can we improve radiology–pathology concordance and reduce interpretative variability for NF cases presenting with suspicious, diagnostically equivocal magnetic resonance imaging findings?

***Findings*** T2 heterogeneity and diffusion measurements are associated with stromal composition and cellularity, while predefined MRI features demonstrated substantial-to-excellent inter-reader agreement.

***Critical relevance statement*** Embedding explicit AHA criteria in the prompt let an 8B model approach 671B-model agreement (38.91–81.52%), supporting standardized text-based workflows without fine-tuning.

**Graphical Abstract:**

**Figure:**
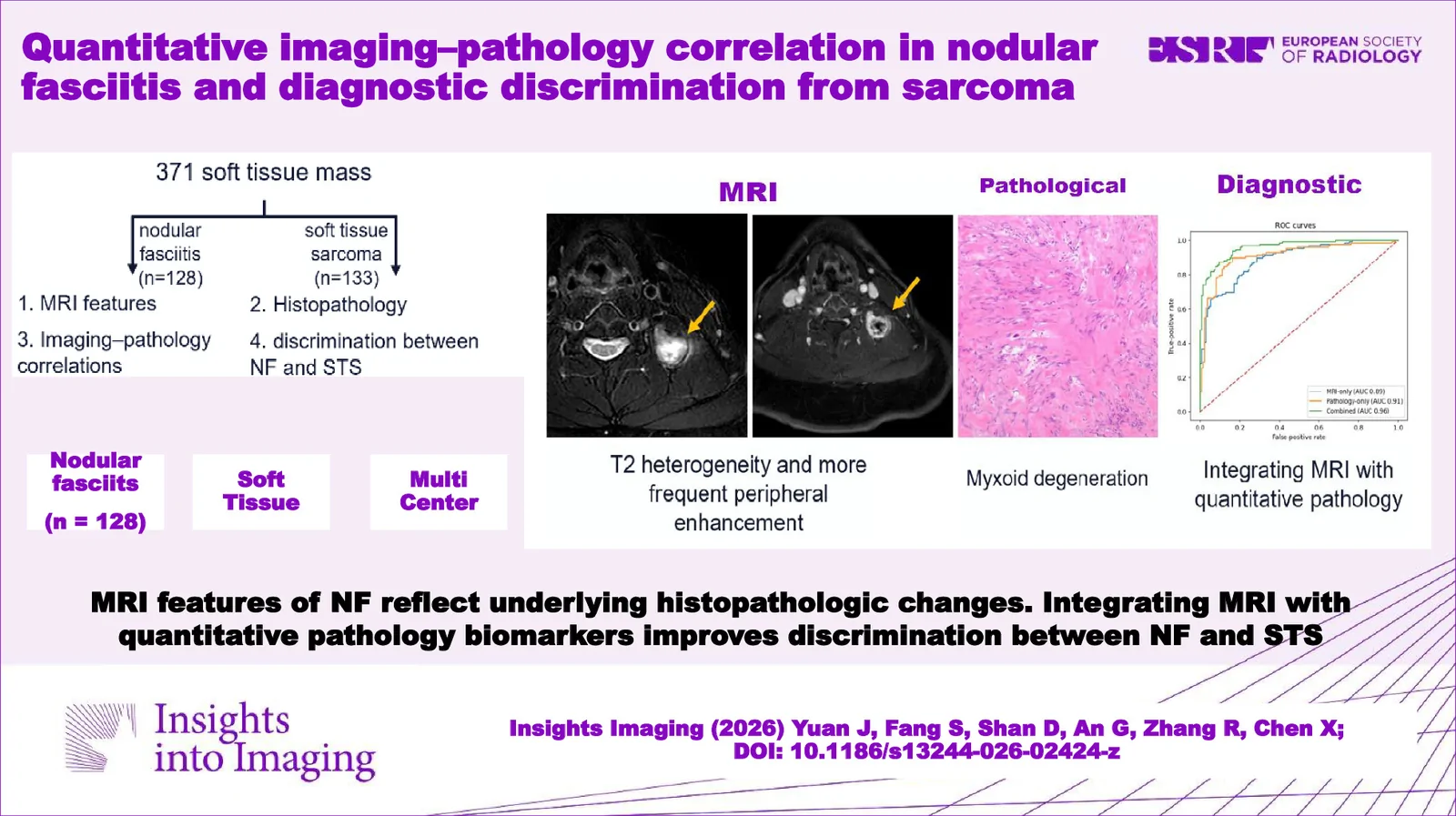

## Introduction

Nodular fasciitis (NF) is a benign, self-limited fibroblastic and myofibroblastic proliferation that typically presents as a rapidly enlarging soft-tissue mass [1, 2]. Although NF demonstrates nonneoplastic behavior, it frequently mimics malignant soft-tissue sarcoma (STS) in both clinical presentation and imaging appearance [3]. This is particularly true when lesions show rapid growth, surrounding edema, and heterogeneous signal intensity on magnetic resonance imaging (MRI) [4, 5]. Consequently, NF remains a well-recognized diagnostic pitfall in musculoskeletal imaging and may lead to overtreatment, including wide excision, unnecessary oncologic evaluation, or referral through sarcoma pathways.

MRI is considered the preferred modality for local characterization of soft-tissue masses because of its superior soft-tissue contrast and its ability to assess fascial relationships, internal composition, and enhancement patterns [6, 7]. The term “imaging features” encompasses the qualitative and quantitative MRI characteristics used for lesion assessment, including lesion location, perilesional edema, signal intensity and heterogeneity, fascial extension, enhancement patterns, and diffusion measurements. Several imaging features have been reported in NF, including a superficial location, perilesional edema, heterogeneous T2-weighted signal intensity, and the “fascial tail sign”. The fascial tail sign refers to linear extension of abnormal signal intensity and/or enhancement along adjacent fascia [8, 9]. However, these imaging descriptors remain largely qualitative, and their diagnostic specificity is limited because substantial overlap exists with STS, particularly myxoid and inflammatory sarcoma subtypes [3, 5]. In addition, the biologic substrates underlying these imaging features have not been systematically quantified. As a result, uncertainty often persists when NF must be distinguished from STS in the preoperative setting, when management decisions are frequently made before definitive surgical pathology is available.

Histopathologic evaluation remains the reference standard for diagnosis. Nevertheless, in routine clinical practice, preoperative decision-making often relies on core-needle biopsy specimens. These samples may be affected by sampling limitations and may not fully reflect stromal heterogeneity. Imaging findings also play an important role in biopsy targeting, surgical planning, and patient counseling. Therefore, a more mechanistic understanding of imaging features and the establishment of reproducible imaging–pathology correlations may provide incremental value beyond categorical pathologic diagnosis alone. Quantitative digital pathology has recently emerged as a robust approach for objectively measuring histologic substrates, including cellular density, collagen content, and the proportion of myxoid stroma. This methodology enables direct comparison with imaging-derived features and supports biologically grounded interpretation of radiologic findings [10, 11].

Accordingly, the purpose of this study was to perform a systematic radiologic–pathologic correlation analysis between predefined imaging features of NF and quantitative histopathologic biomarkers. A secondary objective was to evaluate whether integrating imaging features with quantitative pathology biomarkers provides complementary information for differentiating NF from soft tissue sarcoma (STS) in diagnostically equivocal cases. The intended role of this approach is adjunctive, particularly for radiology–pathology concordance assessment when imaging findings and limited tissue sampling are not fully concordant, rather than replacement of histopathologic diagnosis.

## Materials and methods

### Study design and patient population

This retrospective multicenter study was conducted at Henan Cancer Hospital and Zhongmu County People’s Hospital and was approved by the Medical Ethics Committees of Henan Cancer Hospital and Zhongmu County People’s Hospital (No. 2022-KY-0063-001), and the requirement for written informed consent was waived. Consecutive patients with histopathologically confirmed NF and STS who underwent preoperative MRI between May 2023 and June 2025 were identified through the pathology databases of both participating institutions and cross-referenced with the corresponding radiology information systems and picture archiving and communication systems at each institution. Clinical, imaging, and pathologic data were obtained from the institution at which each patient was diagnosed and treated.

Inclusion criteria were: (a) histopathologic diagnosis of NF or STS established on surgical excision or core-needle biopsy; (b) preoperative MRI performed within 30 days prior to biopsy or surgery; and (c) availability of MRI sequences including T1-weighted imaging, fat-suppressed T2-weighted imaging, diffusion-weighted imaging (DWI) with apparent diffusion coefficient (ADC) maps, and contrast-enhanced T1-weighted imaging.

**Fig. 1:**
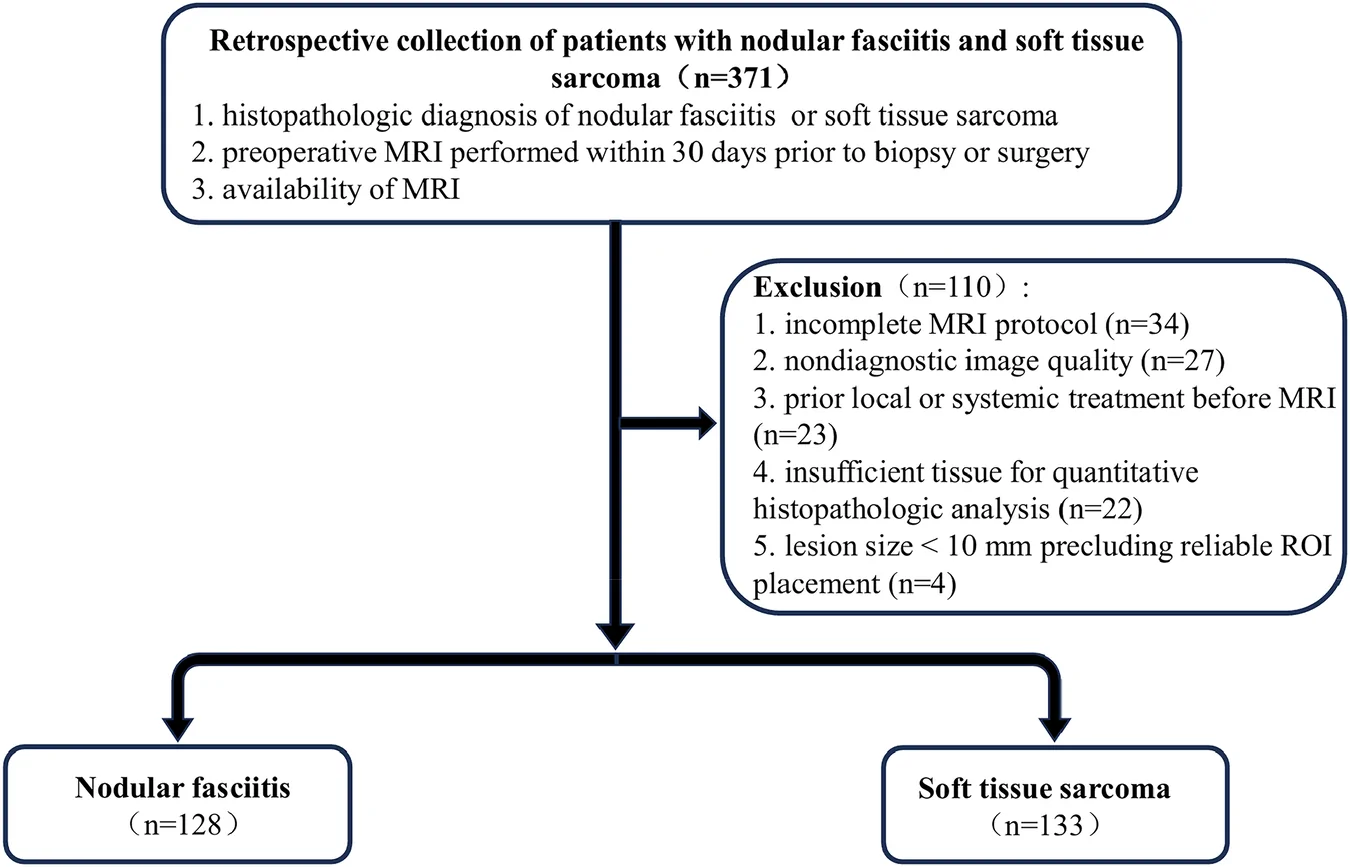
Flowchart for patient recruitment in this study

Exclusion criteria were: (a) incomplete MRI protocol (missing any of the required sequences) (*n* = 34); (b) nondiagnostic image quality due to severe motion or susceptibility artifacts (*n* = 27); (c) prior local or systemic treatment before MRI (surgery, radiotherapy, chemotherapy, or intralesional steroid injection) (*n* = 23); (d) insufficient tissue for quantitative histopathologic analysis (*n* = 22); and (e) lesion size < 10 mm precluding reliable region of interest (ROI) placement (*n* = 4). No formal sample size calculation was performed because this was a retrospective study. All consecutive eligible patients meeting the predefined inclusion and exclusion criteria during the study period were included. The final cohort consisted of 261 patients (NF, *n* = 128; STS, *n* = 133). Patient demographics, lesion location, and maximum lesion diameter were recorded (Fig. 1).

### MRI acquisition

All MRI examinations were performed at Henan Cancer Hospital or Zhongmu County People’s Hospital on two 3-T scanners using dedicated surface coils appropriate for lesion location. Standardized soft-tissue tumor MRI protocols implemented at the two participating institutions included axial and/or coronal T1-weighted imaging, fat-suppressed T2-weighted imaging, DWI with ADC map reconstruction, and contrast-enhanced fat-suppressed T1-weighted imaging.

DWI was acquired using single-shot echo-planar imaging with at least two b values (*b* = 0 and 1000 s/mm²). ADC maps were automatically generated by the scanner software. Intravenous gadolinium-based contrast material (0.1 mmol/kg) was administered at a rate of 2 mL/s, followed by a saline flush. Because lesions arose in multiple anatomical regions, field of view and section thickness were not identical across all patients; instead, acquisition parameters were standardized within predefined protocol ranges according to body region and scanner platform. Detailed acquisition parameters for each scanner are provided in Table 1.

**Table 1:** Detailed MRI acquisition parameters for each scanner

Parameter	Scanner A	Scanner B
Manufacturer	Siemens Healthineers	United Imaging Healthcare
Scanner model	MAGNETOM Skyra	uMR 780
Field strength	3.0 T	3.0 T
Coil	Dedicated surface coil	Dedicated surface coil
T1-weighted imaging		
Sequence type	Fast spin-echo	Fast spin-echo
Imaging plane	Axial ± Coronal	Axial ± Coronal
TR (ms)	650	585
TE (ms)	11	13
Section thickness (mm)	4	4
Intersection gap (mm)	1	1
Field of view (cm)	20	20
Matrix	320 × 256	320 × 256
Fat-suppressed T2-weighted imaging		
Fat suppression technique	SPAIR	SPAIR
Imaging plane	Axial ± Coronal	Axial ± Coronal
TR (ms)	3216	3671
TE (ms)	72	81
Section thickness (mm)	4	4
Intersection gap (mm)	1	1
Field of view (cm)	20	20
Matrix	320 × 256	320 × 256
DWI		
Sequence type	Single-shot echo-planar imaging	Single-shot echo-planar imaging
Imaging plane	Axial	Axial
TR (ms)	4788	4888
TE (ms)	Minimum	Minimum
b values (s/mm²)	0, 1000	0, 1000
Section thickness (mm)	4	4
Intersection gap (mm)	1	1
Field of view (cm)	20	20
Matrix	128 × 128	128 × 128
ADC map generation	Automatic	Automatic
Contrast-enhanced T1-weighted imaging		
Fat suppression technique	SPAIR	SPAIR
Imaging plane	Axial ± Coronal	Axial ± Coronal
TR (ms)	650	585
TE (ms)	11	13
Section thickness (mm)	4	4
Intersection gap (mm)	1	1
Field of view (cm)	20	20
Matrix	320 × 256	320 × 256
Contrast agent dose	0.1 mmol/kg	0.1 mmol/kg
Injection rate (mL/s)	2	2

### Qualitative imaging features

Two fellowship-trained musculoskeletal radiologists (Reader 1: 10 years; Reader 2: 15 years) independently reviewed all MRI examinations. Both readers were blinded to histopathologic diagnosis, pathologic subtype, and clinical information except for lesion location. Disagreements were resolved by consensus in a joint review session; for analyses requiring independent readings, the initial independent reads were used.

Predefined qualitative features included:

1. Fascial tail sign: defined as a linear or tapering extension of abnormal signal intensity and/or enhancement directly contiguous with the lesion margin and tracking along an adjacent fascial plane on at least two consecutive images or on two orthogonal planes. In contrast, perilesional edema was defined as ill-defined T2-hyperintense signal extending into the surrounding soft tissues without a discrete linear fascial extension.
2. T2-weighted signal heterogeneity score: graded on fat-suppressed T2-weighted images using a 4-point scale according to the estimated proportion and conspicuity of mixed internal signal components:
  Score 0: homogeneous signal intensity throughout the lesion without visually appreciable admixture of distinct T2-hyperintense and relatively hypointense components.
  Score 1: mildly heterogeneous signal with mixed internal signal components involving less than 25% of the lesion.
  Score 2: moderately heterogeneous signal with mixed internal signal components involving 25%–50% of the lesion.
  Score 3: markedly heterogeneous signal with mixed internal signal components involving more than 50% of the lesion or with a clearly variegated internal architecture involving most of the lesion.
3. Enhancement pattern: assessed on contrast-enhanced fat-suppressed T1-weighted images and categorized as follows:
  Homogeneous: enhancement involving more than 90% of the enhancing solid portion of the lesion without visually distinct internal variation.
  Heterogeneous: patchy or geographic internal variation in enhancement that did not meet criteria for either homogeneous or rim/peripheral enhancement.
  Rim/peripheral enhancement: predominant peripheral enhancement with a relatively hypoenhancing or nonenhancing central component involving at least one-third of the lesion diameter.
4. Perilesional edema: defined as ill-defined hyperintense signal on fat-suppressed T2-weighted images extending into the surrounding soft tissues beyond the lesion margin without a discrete linear fascial extension.
5. Necrosis: defined as intralesional areas showing very high T2 signal intensity and absent internal enhancement on post-contrast images, interpreted in conjunction with unenhanced T1- and T2-weighted morphology to distinguish necrosis from simple cystic change or myxoid stroma.

### Quantitative diffusion metrics (ADC measurement)

ADC measurements were performed on ADC maps with reference to high-b-value DWI, fat-suppressed T2-weighted images, and contrast-enhanced T1-weighted images. For each lesion, three circular ROIs (20–50 mm², adjusted to lesion size) were placed within the solid-appearing portion of the lesion on the slice or slices showing the largest enhancing solid component. ROI placement preferentially targeted areas corresponding to high signal intensity on high-b-value DWI while avoiding cystic, necrotic, hemorrhagic, and obvious vascular regions. Mean ADC and minimum ADC were recorded, and the lesion-level ADC value was defined as the average of the three ROI measurements.

### Histopathologic review and digital quantification

Quantitative pathology analysis was performed on available tissue specimens from all included patients. Histopathologic specimens and corresponding pathology reports were retrieved from the pathology archives of Henan Cancer Hospital and Zhongmu County People’s Hospital, according to the institution at which each patient was diagnosed and treated. For patients who underwent preoperative core needle biopsy, pathology measurements were derived from biopsy specimens. For patients without preoperative biopsy, quantitative pathology measurements were obtained from surgical resection specimens. Histopathologic diagnosis served as the reference standard. All diagnoses were rendered by board-certified musculoskeletal pathologists according to contemporary WHO classification criteria [12]. The same predefined diagnostic and quantitative assessment criteria were applied to specimens obtained from both participating institutions. For NF, the diagnosis was based on characteristic morphologic findings (plump spindle to stellate fibroblasts/myofibroblasts in a myxoid to collagenous stroma, tissue culture–like growth pattern) with supportive immunohistochemistry when necessary [13]. For STS, histologic subtype and tumor grade were recorded [14].

When both core-needle biopsy and surgical excision specimens were available, the excision specimen was used as the primary reference for diagnosis, whereas biopsy specimens were used for the analysis of biopsy-obtainable quantitative biomarkers reflecting the preoperative setting.

Formalin-fixed paraffin-embedded tissue sections were stained with hematoxylin-eosin (H&E) for routine histologic evaluation. Masson trichrome staining was performed to quantify collagen content. Alcian blue staining was performed when needed to highlight myxoid stroma.

All slides were digitized using a whole-slide scanner at 20× magnification. Slides originating from both participating institutions were analyzed using the same digital pathology workflow and measurement criteria. Quantitative histopathologic biomarkers were derived using digital pathology software (ImageJ). For each case, representative regions were selected to capture the predominant lesion components while avoiding artifact-prone areas (folding, cautery, and crush artifact).

The following quantitative biomarkers were extracted:

1. Cellular density (cells/mm²): automated nuclear detection with manual quality control.
2. Collagen content (% area): percentage of trichrome-positive collagen area relative to total lesion area.
3. Myxoid degeneration (% area): percentage of myxoid stroma area relative to total lesion area on H&E and/or Alcian blue.

### Statistical analysis

Continuous variables were summarized as mean ± standard deviation or median (interquartile range (IQR)) as appropriate, and categorical variables as counts and percentages. Between-group comparisons (NF vs STS) were performed using the Student *t*-test or Mann–Whitney *U*-test for continuous variables and the χ² test or Fisher exact test for categorical variables. Inter-reader agreement was assessed using Cohen’s κ statistics for categorical imaging features and intraclass correlation coefficients (ICCs) (two-way random-effects model) for ADC measurements. Imaging–pathology correlations were assessed using Spearman rank correlation. Multivariable regression models were constructed to evaluate independent associations between imaging features (fascial tail sign, T2 heterogeneity score, enhancement pattern, ADC metrics) and quantitative histopathologic biomarkers (cell density, collagen content, myxoid degeneration), adjusting for lesion size and anatomic location. To evaluate diagnostic performance for differentiating NF from STS, three logistic regression models were developed: (a) imaging features only, (b) quantitative pathology biomarkers only, and (c) combined MRI and pathology biomarkers. Model discrimination was assessed using receiver operating characteristic (ROC) analysis and area under the curve (AUC) with 95% confidence intervals (CIs). Calibration was assessed using calibration plots. Clinical utility was evaluated using decision curve analysis. Internal validation was performed using bootstrap resampling (1000 iterations) to estimate optimism-corrected performance metrics. A two-sided *p* value < 0.05 was considered statistically significant. Statistical analyses were performed using R (version 4.5.1).

## Results

### Patient characteristics

A total of 261 patients were included (NF, *n* = 128; STS, *n* = 133). The NF group was significantly younger than the STS group (median age, 28.0 years [IQR, 23.0–34.0] vs 50.0 years [IQR, 41.0–62.0]; *p* < 0.001). Sex distribution did not differ significantly (male: 73/128 [57.0%] vs 73/133 [54.9%]; *p* = 0.74). Lesions in the NF group were smaller than those in the STS group (median size, 31.1 mm [IQR, 25.3–39.9] vs 53.7 mm [IQR, 43.5–70.1]; *p* < 0.001). Core-needle biopsy specimens were available in 63/128 (49.2%) NF patients and 54/133 (40.6%) STS patients (*p* = 0.173). For patients without preoperative core needle biopsy, quantitative pathology measurements were obtained from surgical resection specimens. Lesion location distribution differed significantly between groups (*p* < 0.001), with NF more frequently located in the head/neck and trunk (Table 2). The STS cohort comprised multiple histologic subtypes according to the current World Health Organization classification, including undifferentiated pleomorphic sarcoma (*n* = 38), liposarcoma (*n* = 32), leiomyosarcoma (*n* = 27), and other less frequent subtypes (*n* = 36). FNCLCC grades were distributed as follows: grade 1, *n* = 28; grade 2, *n* = 45; and grade 3, *n* = 60.

**Table 2:** Baseline patient characteristics of NF and STS groups

Characteristic	NF (*n* = 128)	STS (*n* = 133)	*p* value
Age, y	28.0 (23.0–34.0)	50.0 (41.0–62.0)	< 0.001
Male sex	73 (57.0%)	73 (54.9%)	0.740
Lesion size, mm	31.1 (25.3–39.9)	53.7 (43.5–70.1)	< 0.001
Core-needle biopsy available	63 (49.2%)	54 (40.6%)	0.173
Location			< 0.001
Head/neck	35 (27.3%)	13 (9.8%)	
Trunk	42 (32.8%)	35 (26.3%)	
Upper extremity	23 (18.0%)	26 (19.5%)	
Lower extremity	28 (21.9%)	59 (44.4%)	

### Inter-reader agreement

Inter-reader agreement was substantial to excellent for qualitative imaging features (κ range, 0.66–0.87). Agreement was highest for the fascial tail sign (κ = 0.83) and enhancement pattern (κ = 0.79). ADC measurements showed excellent reliability (ICC = 0.91 for mean ADC and 0.89 for minimum ADC).

### Imaging features in NF vs STS

STS demonstrated greater T2 heterogeneity than NF (median score, 2 [IQR, 2–3] vs 2 [IQR, 1–2], *p* = 0.001). Rim/peripheral enhancement was more common in NF (83/128 [64.8%]) than STS (22/133 [16.5%], overall enhancement distribution *p* < 0.001). Hemorrhage/necrosis was less frequent in NF than in STS (22/128 [17.2%] vs 49/133 [36.8%], *p* < 0.001). Mean ADC was higher in NF (1.40 ± 0.11 × 10⁻³ mm²/s) than STS (1.07 ± 0.14 × 10⁻³ mm²/s, *p* < 0.001), consistent with lower cellular density in NF (Table 3).

**Table 3:** Comparison of imaging features between NF and STS

Characteristic	NF (*n* = 128)	STS (*n* = 133)	*p* value
Fascial tail sign present	36 (28.1%)	31 (23.3%)	0.237
T2 heterogeneity score (0–3)	2.0 (1.0–2.0)	2.0 (2.0–3.0)	0.001
Enhancement pattern			< 0.001
Homogeneous	20 (15.6%)	35 (26.3%)	
Heterogeneous	25 (19.5%)	76 (57.1%)	
Rim/peripheral	83 (64.8%)	22 (16.5%)	
Perilesional edema present	86 (67.2%)	99 (74.4%)	0.221
Necrosis present	22 (17.2%)	49 (36.8%)	< 0.001
Mean ADC (×10⁻³ mm²/s)	1.40 ± 0.11	1.07 ± 0.14	< 0.001
Minimum ADC (×10⁻³ mm²/s)	1.23 ± 0.13	0.85 ± 0.17	< 0.001

### Quantitative histopathologic biomarkers

**Fig. 2:**
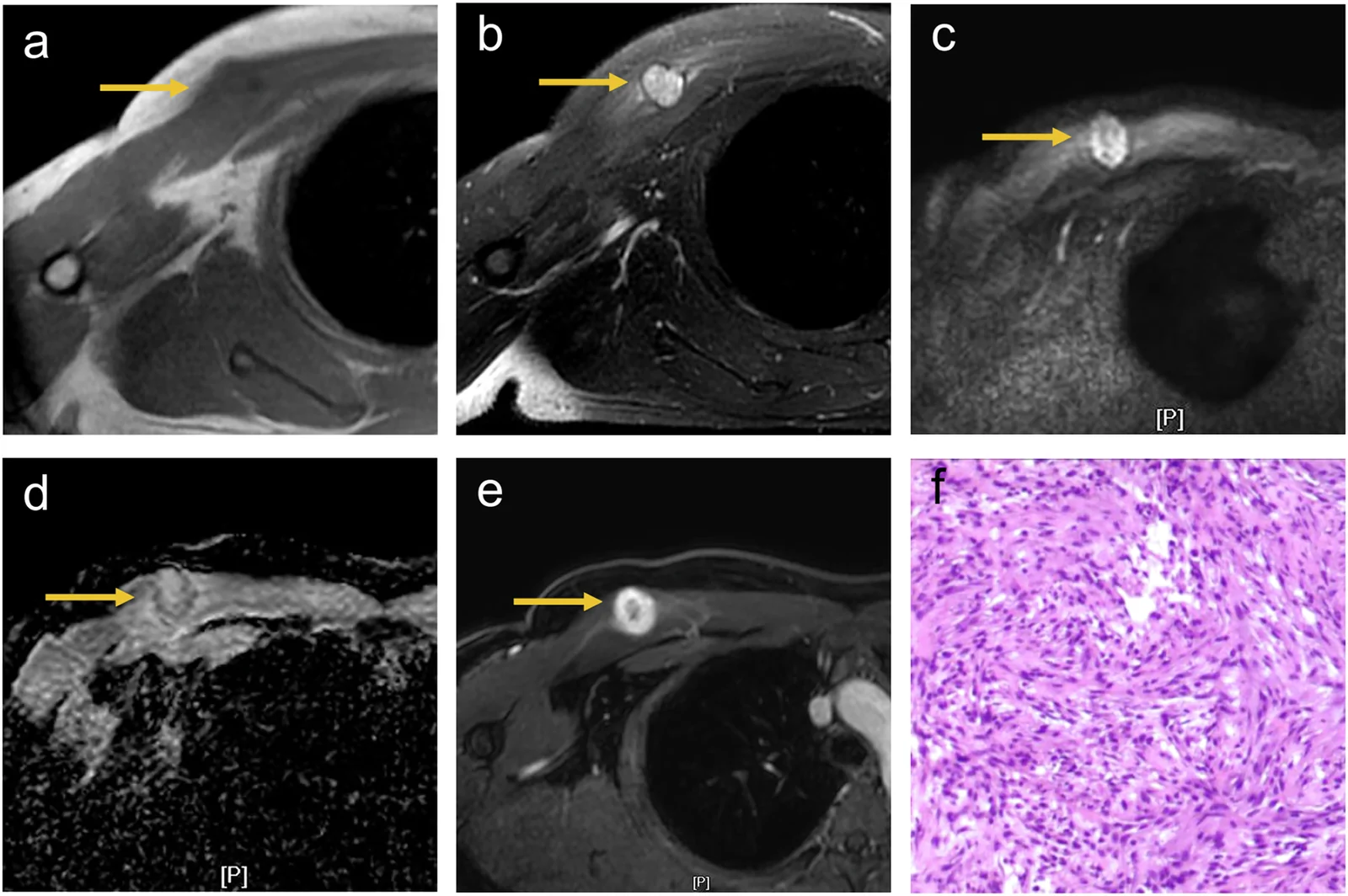
NF in the right chest wall in a 27-year-old man. **a** Axial T1-weighted image shows a well-circumscribed round mass with low signal intensity in the right pectoralis major muscle (arrow). **b** Axial fat-suppressed T2-weighted image demonstrates heterogeneous hyperintensity within the lesion (arrow). **c** Diffusion-weighted image (*b* = 1000 s/mm²) shows intermediate-to-high signal intensity within the lesion (arrow). **d** ADC map shows a mean ADC value of 1.33 × 10⁻³ mm²/s without marked diffusion restriction (arrow). **e** Contrast-enhanced fat-suppressed T1-weighted image demonstrates peripheral (rim) enhancement (arrow), a characteristic enhancement pattern in NF. **f** Photomicrograph (hematoxylin-eosin stain, original magnification ×200) demonstrates a proliferation of spindle-shaped fibroblasts/myofibroblasts arranged in short fascicles within a myxoid stroma, consistent with NF

**Fig. 3:**
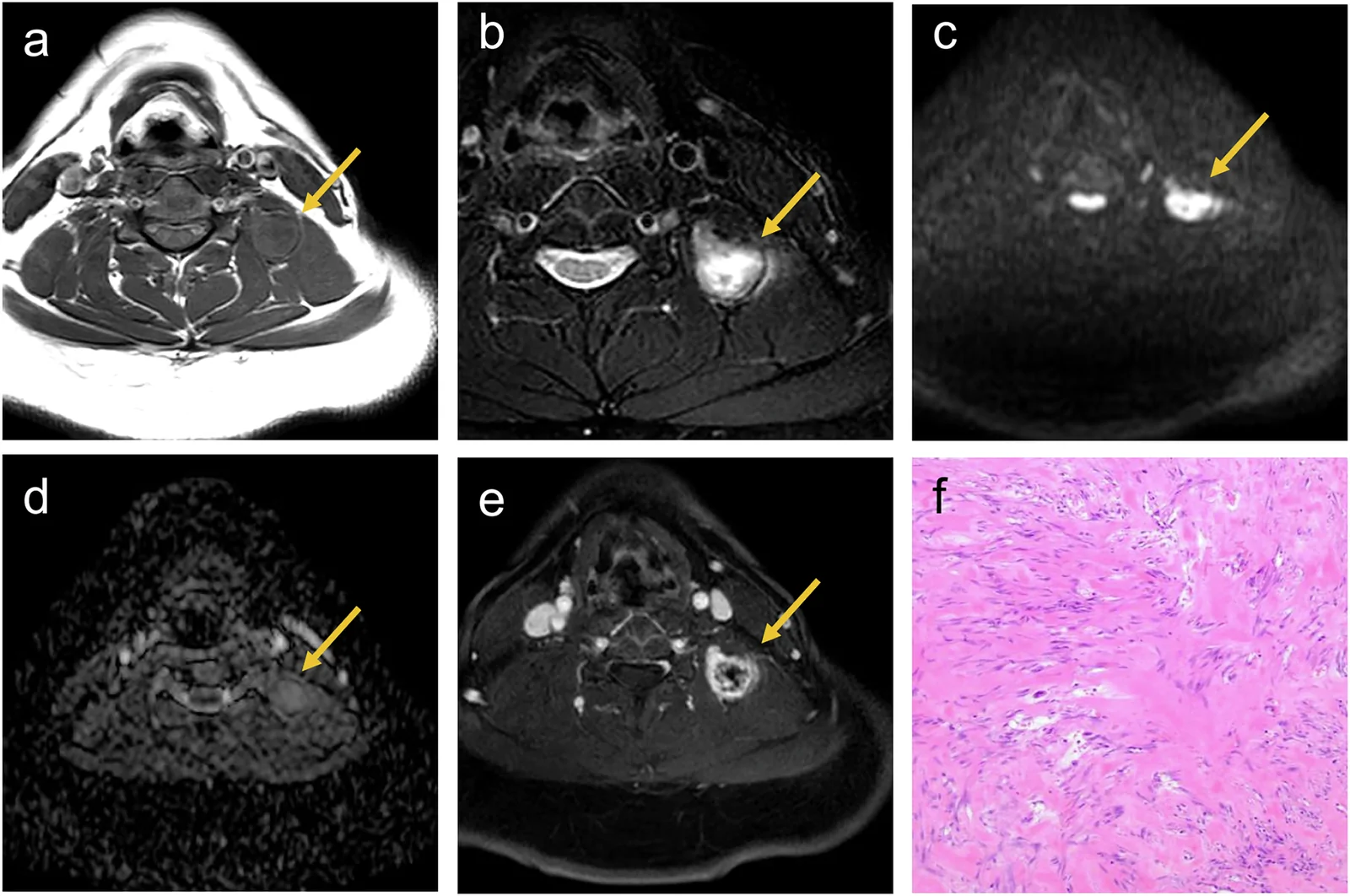
NF in the left neck in a 26-year-old man. **a** Axial T1-weighted image shows a well-defined, round soft-tissue mass in the left neck (arrow) with low signal intensity relative to skeletal muscle. **b** Axial fat-suppressed T2-weighted image demonstrates marked hyperintensity of the lesion (arrow). **c** Diffusion-weighted image (*b* = 1000 s/mm²) shows high signal intensity within the lesion (arrow). **d** ADC map shows a mean ADC value of 1.51 × 10⁻³ mm²/s without marked diffusion restriction (arrow). **e** Contrast-enhanced fat-suppressed T1-weighted image demonstrates prominent peripheral (rim) enhancement of the lesion (arrow). **f** Photomicrograph (hematoxylin-eosin stain; original magnification, ×200) shows spindle-shaped fibroblasts/myofibroblasts arranged in short fascicles within abundant myxoid stroma, consistent with NF

NF demonstrated lower cellular density and higher myxoid degeneration than STS. Cellular density was 1909.3 (1509.8–2217.5) cells/mm² in NF vs 2559.6 (2159.4-2927.4) cells/mm² in STS (*p* < 0.001). Myxoid degeneration was 43.8% (30.8%–55.5%) in NF vs 24.5% (15.3%–32.5%) in STS (*p* < 0.001). Collagen content was modestly higher in NF (21.0% [15.0%–29.4%]) than in STS (14.4% [8.0%–21.7%], *p* < 0.001) (Table 4, and Figs. 2 and 3).

**Table 4:** Comparison of quantitative pathology biomarkers between NF and STS

Characteristic	NF (*n* = 128)	STS (*n* = 133)	*p* value
Cellular density (cells/mm²)	1909.3 (1509.8–2217.5)	2559.6 (2159.4–2927.4)	< 0.001
Collagen content (% area)	21.0 (15.0–29.4)	14.4 (8.0–21.7)	< 0.001
Myxoid degeneration (% area)	43.8 (30.8–55.5)	24.5 (15.3–32.5)	< 0.001

### Imaging–pathology correlation in NF

**Fig. 4:**
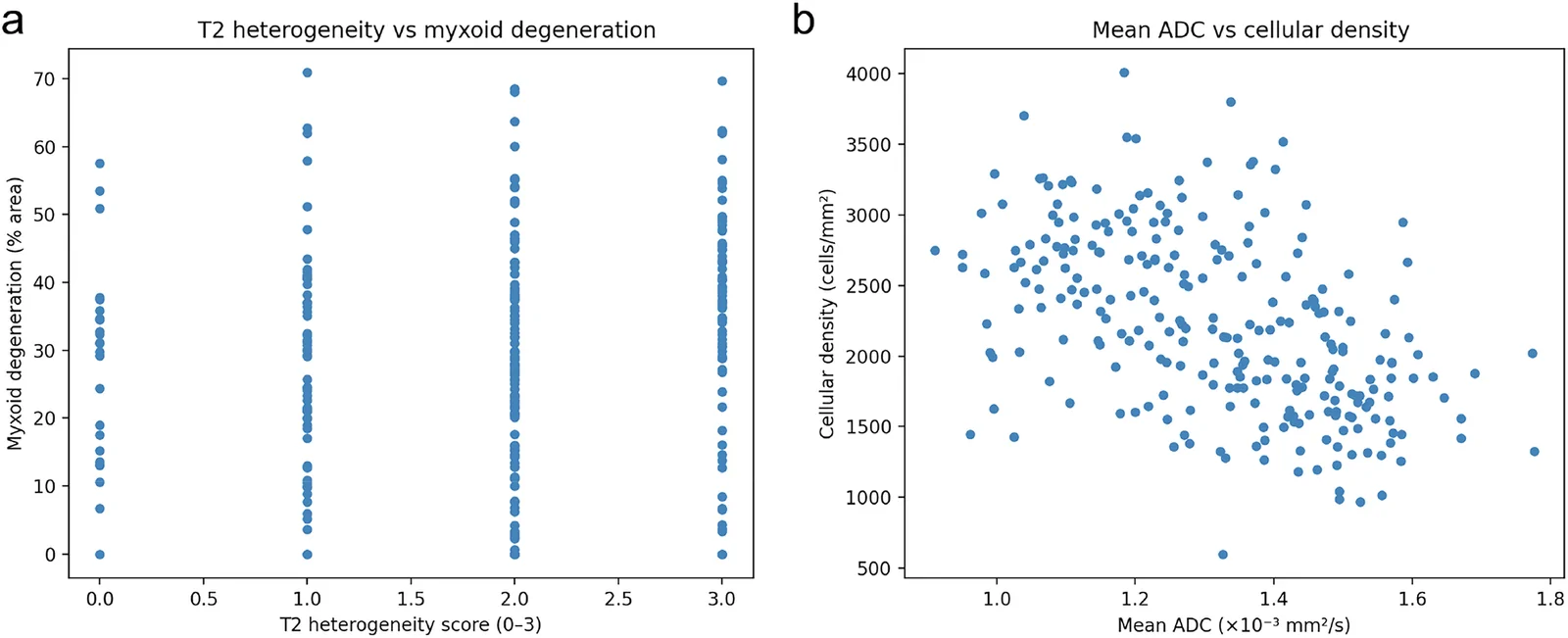
Quantitative imaging–pathology correlations. **a** Scatterplot shows the association between the T2-weighted heterogeneity score (0–3) and the proportion of myxoid degeneration (% area) on digital pathology. **b** Scatterplot shows the inverse association between mean ADC (×10⁻³ mm²/s) and cellular density (cells/mm²), supporting diffusion imaging as a surrogate marker of microscopic cellularity

T2 heterogeneity score correlated positively with myxoid degeneration (Spearman ρ = 0.52, *p* < 0.001) and negatively with collagen content (ρ = −0.28, *p* < 0.001). Mean ADC showed a strong inverse correlation with cellular density (ρ = −0.63, *p* < 0.001) (Fig. 4).

### Diagnostic performance

**Fig. 5:**
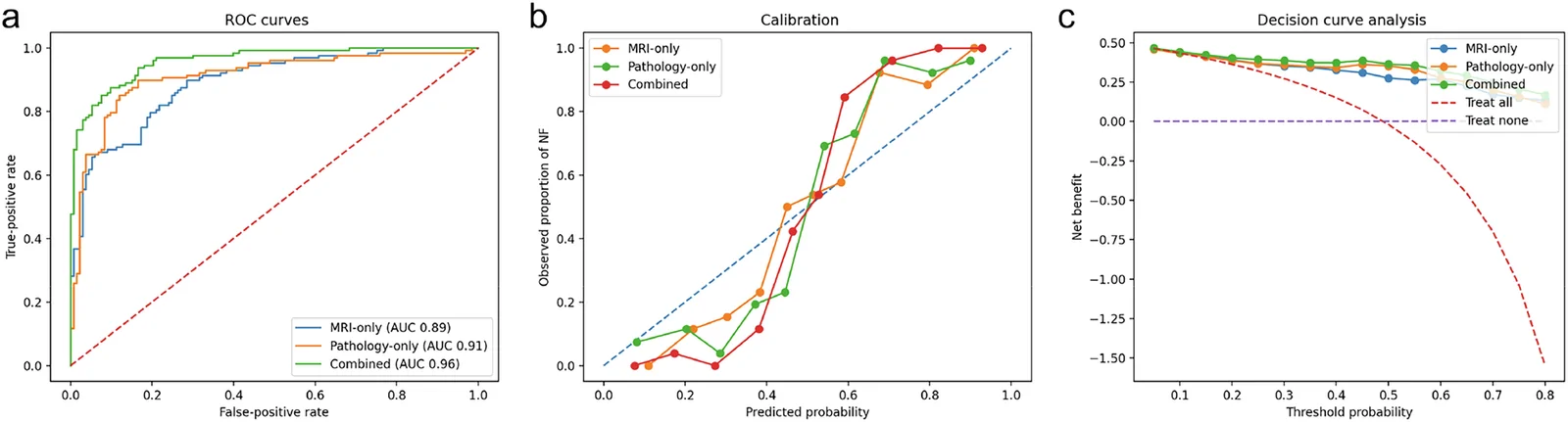
Diagnostic performance and clinical utility. **a** ROC curves for differentiating NF from soft-tissue sarcoma using an MRI-only model (AUC, 0.89), a quantitative pathology–only model (AUC, 0.91), and a combined model (AUC, 0.96). **b** Calibration curves demonstrate agreement between predicted probabilities and observed outcomes across risk strata. **c** Decision curve analysis shows higher net benefit for the combined model across clinically relevant threshold probabilities compared with MRI-only and pathology-only models, as well as “treat all” and “treat none” strategies

Diagnostic performance is shown in Fig. 5. The MRI-only model achieved an AUC of 0.89, whereas the pathology-biomarker model achieved an AUC of 0.91. The combined model integrating imaging features and quantitative pathology biomarkers demonstrated the highest discrimination (AUC 0.96). Calibration plots showed good agreement between predicted and observed probabilities. Decision curve analysis indicated higher net benefit for the combined model across clinically relevant threshold probabilities compared with MRI-only and pathology-only models. Bootstrap internal validation (1000 resamples) yielded a mean AUC of 0.960 (95% CI: 0.938–0.978) for the combined model.

## Discussion

In this study, we demonstrated quantitative imaging–pathology correlations in NF and evaluated their diagnostic relevance in comparison with soft-tissue sarcoma. The findings should not be interpreted as identifying new MRI signal mechanisms, because the relationships between T2 signal, stromal composition, diffusion metrics, and cellularity are already well established in musculoskeletal imaging [8, 9, 15]. Rather, the main contribution of this study is the disease-specific application of these principles to NF, a benign lesion that frequently mimics soft-tissue sarcoma.

Although soft-tissue sarcoma showed greater T2 heterogeneity than NF at the group level, the degree of T2 heterogeneity within NF was biologically interpretable [16, 17]. Specifically, T2 heterogeneity correlated positively with myxoid degeneration and negatively with collagen content, suggesting that heterogeneous signal in NF may reflect benign stromal admixture rather than malignant necrosis or aggressive tumor heterogeneity [18]. This distinction is clinically relevant because heterogeneous T2 signal is often regarded as a suspicious imaging feature in soft-tissue mass evaluation.

Diffusion findings should also be interpreted cautiously. Mean ADC was higher in NF than in soft-tissue sarcoma and showed a strong inverse association with cellular density within NF, supporting the concept that diffusion restriction reflects microscopic cellularity rather than tumor grade per se [6, 19–21]. NF may exhibit relatively high cellularity and inflammatory components and may therefore demonstrate high signal intensity on DWI. Nevertheless, the overall ADC distribution remained higher in NF than in STS in this cohort. Diagnostic discrimination was improved when ADC values were interpreted in combination with morphologic and enhancement features [22].

The combined MRI–pathology model is not intended to replace histopathologic diagnosis. Its potential clinical role is in selected post-biopsy cases where diagnostic uncertainty remains, such as limited biopsy sampling, equivocal biopsy results, or discordance between imaging findings and pathology [23]. In such cases, quantitative pathology biomarkers consistent with NF may help contextualize otherwise suspicious imaging features, thereby supporting multidisciplinary diagnostic confidence and consideration of follow-up or limited excision. Conversely, persistent inconsistency between imaging and pathology findings may prompt repeat targeted biopsy or continued management through a sarcoma pathway.

Previous studies have described imaging characteristics of NF, including superficial location, rapid growth, perilesional edema, and occasional fascial extension [1, 24]. However, most prior reports were qualitative or based on small cohorts, and mechanistic radiologic–pathologic correlations were limited. By integrating pathology quantification with standardized MRI features in a relatively large cohort, the present study extends the existing literature. Quantitative evidence was provided linking imaging features to specific histologic substrates, and incremental diagnostic value was demonstrated through multimodal integration.

Several strengths should be acknowledged. First, the cohort size was relatively large for NF and included a clinically relevant STS comparator group. Second, imaging features were predefined and demonstrated substantial to excellent inter-reader agreement, supporting reproducibility. Finally, model performance and clinical utility were assessed using calibration analysis, decision curve analysis, and bootstrap internal validation.

This study also has limitations. First, although this was a retrospective multicenter study involving two institutions, selection bias cannot be excluded because patient inclusion depended on the availability of preoperative MRI and histopathologic confirmation. In addition, the findings were derived from only two centers and therefore may not fully reflect patient populations and imaging practices at other institutions. Second, MRI examinations were performed on two 3-T scanners. Although institutional protocols were standardized and acquisition parameters were detailed in the supplemental material, scanner-related variability may have influenced diffusion measurements. Third, imaging–pathology correlations were performed at the lesion level rather than through precise spatial coregistration, which may have attenuated correlation strength. Fourth, the control group included heterogeneous sarcoma subtypes. Although this reflects real-world diagnostic practice, subtype-specific analyses may be warranted. In addition, no a priori sample size calculation was performed because of the retrospective study design, and the available sample was determined by the number of eligible cases during the study period.

## Conclusion

Quantitative MRI–pathology correlation analysis demonstrated that key imaging features of NF reflect measurable stromal and cellular substrates. The clinical value of this approach lies in supporting radiology–pathology concordance assessment in selected diagnostically equivocal cases, rather than replacing histopathologic diagnosis.

## Supplementary information

ELECTRONIC SUPPLEMENTARY MATERIAL

## Data Availability

Data available on request: the data underlying this article will be shared on reasonable request to the corresponding author.

## Abbreviations

ADC: Apparent diffusion coefficient
AUC: Area under the curve
DWI: Diffusion-weighted imaging
H&E: Hematoxylin and eosin
ICC: Intraclass correlation coefficient
IQR: Interquartile range
MRI: Magnetic resonance imaging
NF: Nodular fasciitis
ROC: Receiver operating characteristic
ROI: Region of interest
STS: Soft tissue sarcoma
