# Supplementary material for: Quantitative imaging–pathology correlation in nodular fasciitis and diagnostic discrimination from sarcoma
Source: Insights Imaging. 2026 Nov 2;17:274. doi: 10.1186/s13244-026-02424-z (PMC13728007; doi:10.1186/s13244-026-02424-z)
Supplement: Supplementary file 1 [file 13244_2026_2424_MOESM1_ESM.pdf]

Quantitative Imaging–Pathology Correlation in Nodular Fasciitis and Diagnostic Discrimination from Sarcoma

ELECTRONIC SUPPLEMENTARY MATERIAL

Standardized Institutional MRI Acquisition Protocol

All MRI examinations were performed using two 3.0-T scanners (MAGNETOM Skyra, Siemens Healthineers; uMR 780, United Imaging Healthcare) equipped with dedicated surface coils selected according to lesion location.

To ensure consistency across scanners and anatomical regions, institutional soft-tissue tumor imaging protocols were standardized and harmonized prior to study initiation. Protocol standardization included predefined ranges for sequence type, spatial resolution, and acquisition parameters. Although minor variations in field of view and section thickness were permitted to accommodate lesion size and anatomical location, all parameters were maintained within institutionally defined limits to ensure comparability.

The standardized imaging protocol consisted of:

- Axial and/or coronal T1-weighted imaging
- Fat-suppressed T2-weighted imaging
- Diffusion-weighted imaging (DWI) with apparent diffusion coefficient (ADC) map reconstruction
- Contrast-enhanced fat-suppressed T1-weighted imaging

Diffusion-Weighted Imaging Protocol

DWI was performed using single-shot echo-planar imaging on both scanners. At least two b values (0 and 1000 s/mm^2) were applied in all examinations. Echo time (TE) was minimized to optimize signal-to-noise ratio. ADC maps were automatically generated using vendor-provided software based on a mono-exponential model.

Contrast Administration Protocol

Intravenous gadolinium-based contrast material was administered at a standardized dose of 0.1 mmol/kg body weight using a power injector at a rate of 2 mL/s, followed by a saline flush. Post-contrast imaging was performed using fat-suppressed T1-weighted sequences in axial and/or coronal planes.

Scanner-Specific MRI Acquisition Parameters

Detailed acquisition parameters for each scanner are summarized below.

Scanner Platforms

- **Scanner A**: MAGNETOM Skyra (Siemens Healthineers)
- **Scanner B**: uMR 780 (United Imaging Healthcare)
- Field strength: 3.0 T (both systems)
- Coil: Dedicated surface coil

Table. Detailed MRI acquisition parameters for each scanner

Parameter	Scanner A	Scanner B
Manufacturer	Siemens Healthineers	United Imaging Healthcare
Scanner model	MAGNETOM Skyra	uMR 780
Field strength	3.0 T	3.0 T
Coil	Dedicated surface coil	Dedicated surface coil
**T1-weighted imaging**		
Sequence type	Fast spin-echo	Fast spin-echo
Imaging plane	Axial ± Coronal	Axial ± Coronal
TR (ms)	650	585
TE (ms)	11	13
Section thickness (mm)	4	4
Intersection gap (mm)	1	1
Field of view (cm)	20	20
Matrix	320 × 256	320 × 256
**Fat-suppressed T2-weighted imaging**		
Fat suppression technique	SPAIR	SPAIR
Imaging plane	Axial ± Coronal	Axial ± Coronal
TR (ms)	3216	3671

TE (ms)	72	81
Section thickness (mm)	4	4
Intersection gap (mm)	1	1
Field of view (cm)	20	20
Matrix	320 × 256	320 × 256
**DWI**		
Sequence type	Single-shot echo-planar imaging	Single-shot echo-planar imaging
Imaging plane	Axial	Axial
TR (ms)	4788	4888
TE (ms)	Minimum	Minimum
b values (s/mm²)	0, 1000	0, 1000
Section thickness (mm)	4	4
Intersection gap (mm)	1	1
Field of view (cm)	20	20
Matrix	128 × 128	128 × 128
ADC map generation	Automatic	Automatic
**Contrast-enhanced T1-weighted imaging**		
Fat suppression technique	SPAIR	SPAIR
Imaging plane	Axial ± Coronal	Axial ± Coronal
TR (ms)	650	585
TE (ms)	11	13
Section thickness (mm)	4	4
Intersection gap (mm)	1	1
Field of view (cm)	20	20
Matrix	320 × 256	320 × 256
Contrast agent dose	0.1 mmol/kg	0.1 mmol/kg
Injection rate (mL/s)	2	2
